# Employing computational fluid dynamics technique for analyzing the PACK-1300XY with methanol and isopropanol mixture

**DOI:** 10.1038/s41598-022-10590-5

**Published:** 2022-04-21

**Authors:** Y. Cao, H. A. Dhahad, A. Khandakar, M. E. H. Chowdury, M. A. Ayari, S. M. Alizadeh, B. Vaferi

**Affiliations:** 1 School of Mechatronic Engineering, Xi’an Technological University, Xi’an, 710021 China; 2 Mechanical Engineering Department, University of Technology, Baghdad, Iraq; 3 Electrical Engineering Department, Qatar University, Doha, 2713, Qatar; 4 Civil and Architectural Engineering Department, College of Engineering, Qatar University, Doha, 2713, Qatar; 5 Petroleum Engineering Department, Australian College of Kuwait, West Mishref, Kuwait; 6 Department of Chemical Engineering, Shiraz Branch, Islamic Azad University, Shiraz, Iran

**Keywords:** Energy science and technology, Engineering, Mathematics and computing

## Abstract

In this study, an innovative wire gauze structured packing, namely PACK-1300XY with a specific surface area of 1300 m^2^/m^3^ has been characterized by performing computational fluid dynamics (CFD) approach. Indeed, different features of this packing (height equivalent to a theoretical plate, wet/dry pressure drop, and mass transfer efficiency) were analyzed by analyzing the flow regime using the three-dimensional CFD approach with the Eulerian–Eulerian multiphase scenario. The results showed the mean relative deviation of 16% (for wet pressure drop), 14% (for dry pressure drop), and 17% (for mass transfer efficiency) between the CFD predictions and experimental measurements. These excellent levels of consistency between the numerical findings and experimental observations approve the usefulness of the CFD-based approach for reliable simulation of separation processes.

## Introduction

Separation technologies, like ionic liquid-based^1,2^ and microvaiwe assisted^3^ absorption, adsorption^4–7^, membrane^8^, circulating purification^9^, and fluidizedization^10–12^ have a wide ranges of application in different applications. The distillation column is likely the most well-known process to separate either feedstock and product stream by considering the relative volatility^13–15^. The distillation column can easily handle a counter-current flow regime between gas and liquid streams^16^. The distillation columns equipped with the tray^17^ and packing^18^ are two main categories in this regard. The latter is divided into two random and structured packings^19^.

Because the structure packing has a wide range of benefits, including providing low-pressure drop, having high separation efficiency, and being adaptable with the different operations^20^, it has been selected for the large-scale separation towers^21^ and domestic chemical industries. In order to elaborate on the separation performance of the chemical procedures, one kind of structured packings, i.e., corrugated sheet packing, is popular^22^. Their material could be metals, ceramics, and plastics^23^ arranged side by side with reverse canal directions. Therefore, their porosity is about ninety percent, with an attractive specific surface area ranging from 250 to 750 m^2^/m^3^^24^.

The dry and wet pressure drops in the distillation columns are associated with the gas phase and the counter-current gas–liquid stream across the column height, respectively^25^. Moreover, the height equivalent to a theoretical plate (HETP) and liquid hold up are other essential characteristics of a packing^26,27^. Moreover, the separation efficiency for the packed bed has been defined via the HETP-value, which shows the mass transfer of packings. It should be noted that there is a proportional relation between these parameters^28^.

Among the numerical studies for the fluid flow throughout the packings, computational fluid dynamic (CFD) is an efficient method to reduce the economic expense of experimental measurements^29–32^. Moreover, another benefit of this computational method is the capability of estimation for the effect of diverse factors^33–35^. To serve this purpose, several researchers developed precise discretization methods and the solution algorithms, i.e., providing an effective computational scheme, to introduce an accurate model for the simulation of the flow in the packings which the combination of in-house scripts and software would be used to this end^36^.

In recent years, an enrichment of components at the gas–liquid interface^37,38^ on the atomistic scale has an important influence on mass transfer mechanisms^39–41^. Moreover, several CFD-based studies have investigated the multiphase flow behavior of differently structured packings^42–45^. Moreover, the study of the empirical representation for commercial use of corrugated packings sheets has been presented in other computational works^46,47^.

Amini et al. fabricated different wire gauze structured packings with the specific surface area range from 860 to 2100 m^2^/m^3^^48–50^. They observed a low-pressure drop for both dry and wet streams, low HETP, high specific surface area, and high liquid holdup for the fabricated packings. They also investigated the effect of different operational conditions on the performance of these structured packings employing the multiphase flow simulations.

Liu and co-workers performed the three-dimensional single-phase CFD simulations to study the behavior of the rotary packed bed (RPB) equipped with the wire mesh structured packing from stainless steel^51^. They studied the pressure, pressure drop, and gas flow profiles around the packing as a function of gas flow rate and rotational velocity. Moreover, by using the analysis of the gas flow behavior and pressure distribution and CFD method, they studied the geometry optimization of packing and RPB. It should be noted that several researchers focused on the simulation of the two-phase flow^52^ and mass transfer in the interface of the structured packings^53^.

Some efforts were also made to modify the current models and increase the calculation accuracy of the liquid holdup and pressure drop in the structured packings^54^ and mass transfer evaluation^55^. Van Baten and Krishna investigated the mass transfer behaviors of the gas and liquid in a katapak-S packing^54^. They reported a good agreement between their results and the theoretical correlation presented by Viva et al.^56^. In addition, Manh et al. by using CFD simulation calculated the dry and wet pressure drop and mass transfer efficiency in the new structured packing^57^. The authors proposed a model near to the real geometry. The simulation results were in good agreement with experimental data^57^.

This computational study aims to characterize a newly fabricated wire gauze structured packing employing the CFD approach. Another goal is the evaluation of this packing under different operating conditions. Furthermore, the CFD technique helps analyze the HETP, mass transfer efficiency, and wet and dry pressure of this new structured packing. In this approach, CFD characterizes the new structured packing (PACK-1300XY) using the isopropanol/methanol mixture.

## Simulation details

### Hydrodynamics

The flow geometry of the Newtonian/incompressible fluid inside the column in the isothermal condition has been simulated using the multiphase flow Eulerian/Eulerian model^58^. It should be noted that all the fluid-related characteristics are assumed to have constant behavior across the column height.

Moreover, the following equations are used to calculate the mass and momentum conservation laws in a volume-averaged mode, respectively^59–62^.

1 $$\frac{\partial }{\partial t}\left( {\gamma_{\alpha } \rho_{\alpha } } \right) + \nabla \left( {\gamma_{\alpha } \rho_{\alpha } U_{\alpha } - {\Gamma }_{\alpha } \nabla \gamma_{\alpha } } \right) = 0\quad \alpha = L, G$$

2 $$\frac{\partial }{\partial t}\left( {\gamma_{\alpha } \rho_{\alpha } U_{\alpha } } \right) + \gamma_{\alpha } \left\{ {\rho_{\alpha } {\text{U}}_{\alpha } U_{\alpha } - \left[ {\mu_{e\alpha } \left( {\nabla U_{\alpha } + \left( {\nabla U_{\alpha } } \right)^{T} } \right)} \right]} \right\} = \gamma_{\alpha } \left( {B_{\alpha } \nabla {\text{P}}} \right) - F_{\alpha } \quad \alpha = L, G$$

where *ρ*, ${\upgamma }$, *μ* and *U* are the fluid density, the occupied volume fraction by phases, the dynamic viscosity, and the interstitial velocity, respectively. In addition to these parameters, Γ indicates the volume fraction dispersion factor, $\mu_{e}$ and $F_{\alpha }$ designate the effective viscosity and the interfacial drag force, respectively.

### Mass transfer behavior

The following equation was used to calculate the mass transfer relation:

3 $$\begin{aligned} & \frac{\partial }{\partial t}\left( {\gamma_{\alpha } \rho_{\alpha } Y_{i\alpha } } \right) + \nabla \left[ {\gamma_{\alpha } \left( {\rho_{\alpha } U_{\alpha } Y_{i\alpha } - {\Gamma }_{\alpha } \nabla Y_{i\alpha } } \right)} \right] = \mathop \sum \limits_{{\begin{array}{*{20}c} {\beta = 1,} \\ {\beta \ne \alpha } \\ \end{array} }}^{N} m_{\alpha \beta }^{i} { } \\ & \quad \quad \alpha = 1, \ldots ,{ }N,\quad \beta = 1, \ldots ,{ }N_{C} \\ \end{aligned}$$

where *N* and *N*_C_ show numbers of phase and numbers of phase ingredients, respectively. Furthermore, *Y* is the weight fraction of the *i*th ingredient in a specific phase. $m_{\alpha \beta }^{i}$ indicates the transferred mass per unit volume per unit time of the *i*th ingredient from phase $\beta$ to phase $\alpha$. It is obvious that the weight fraction must satisfy the following equation:

4 $$\mathop \sum \limits_{{{\text{i}} = 1}}^{{N_{c} }} {\text{Y}}_{{\text{i}}} { } = 1$$

Equation (5) presents the theoretical basis of calculating the $m_{\alpha \beta }^{i}$ using the two-film theory. This equation states that the summation of the gas ($\gamma_{G}$) and liquid ($\gamma_{L}$) phase volume fractions are equal to one:

5 $$\gamma_{G} + \gamma_{L} = 1$$

where

6 $$\gamma_{G} = \frac{{V_{G} }}{{V_{G} + V_{L} }}$$

7 $$\gamma_{L} = \frac{{V_{L} }}{{V_{G} + V_{L} }}$$

### Mass transfer in the interphase region

It is necessary to highlight that the interphase mass transfer, which several researchers have adequately described, is used in this regard^63^.

### Mass transfer efficiency

A Series of theoretical stages were used to predict the HETP value as follows^63^:

8 $${\text{HETP}} = \left[ {\frac{\ln \lambda }{{\lambda - 1}}} \right]H_{OG}$$

9 $${\text{H}}_{{{\text{OG}}}} = H_{G} + \lambda H_{L} = \frac{{U_{GS} }}{{k_{G} a_{e} }} + \lambda \frac{{U_{LS} }}{{k_{L} a_{e} }}$$

10 $$\lambda = \frac{m}{L/G} = \frac{\alpha }{{\left[ {1 + \left( {\alpha - 1} \right)x_{A} } \right]^{2} }}.\frac{G}{L}$$

where $\lambda$ shows the equilibrium per operation line slopes. Other parameters (k_G_, k_L_, and a_e_) are directly achieved from the proposed model by Bravo et al.^16^.

### Turbulent flow

Because we deal with complication flow characteristics inside the structured packing, utilizing the turbulent model to obtain reliable results is inevitable. Therefore, the literature review approved that the ‘Standard’ k − ω model proposed by Wilcox is a suitable turbulent model for our considered case^64,65^. Moreover, this model provides a reliable prediction for the flow characteristic by considering the near-wall treatment for a small Reynolds number^66–69^.

One of the advantages of the k–ω formulation is the near wall treatment for low-Reynolds number computations. The model does not involve the complex non-linear damping functions required for the k–ε model and is therefore more accurate and more robust. A low-Reynolds k–ε model would typically require a near wall resolution of y^+^ < 0.2, while a low-Reynolds k–ω model would require at least y^+^ < 2. In industrial flows, even y^+^ < 2 cannot be guaranteed in most applications and for this reason; a near wall treatment needs to be used. It allows for a smooth shift from a low-Reynolds number form to a wall function formulation. The k–ω model assumes that the turbulence viscosity is linked to the turbulence kinetic energy and turbulent frequency via the relation:

11 $$\mu_{t} = \rho \frac{k}{\omega }$$

## The packing geometry and numerical simulation

**Figure 1:**
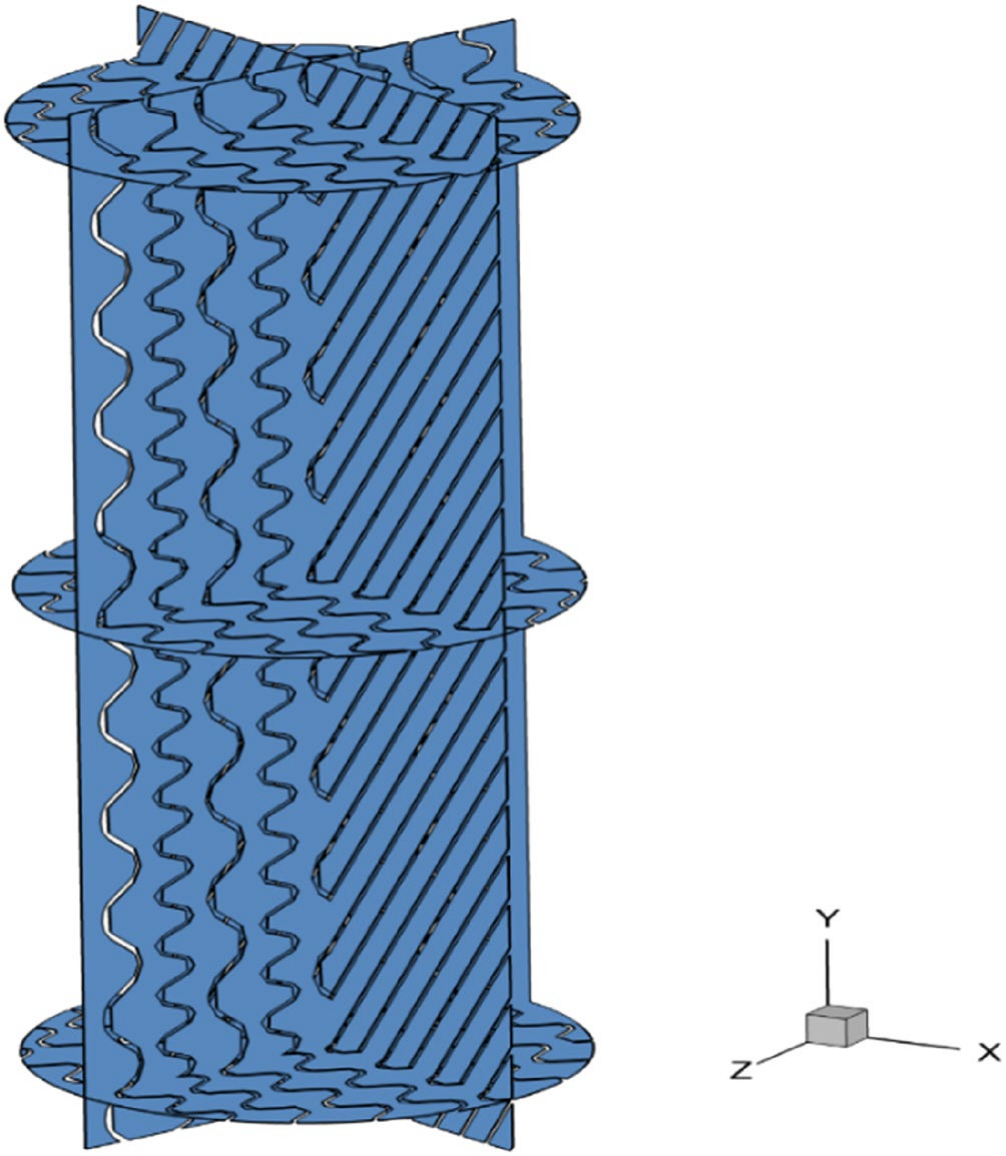
The simulated geometries of the PACK-1300XY.

Among the steps of CFD simulations, the most crucial one is related to preparing a suitable grid with acceptable precision^70,71^. For serving this purpose, a well-defined three-dimensional geometry with a proper number of elements is first constructed. Moreover, in this study, twelve packing sheets are simulated inside a cylindrical system. To better understand, the computational geometry of the structured packing have been illustrated in Fig. 1.

This study uses a wire gauze made from metal sheets to fabricate the structured packing known as PACK-1300XY. This packing was introduced by Dinh Manh et al.^72^. Also in this research, we used the experimental data that was published^72^. The specific area and porosity are 1300 m^2^/m^3^ and 0.91, respectively. It should be stated that this structured packing is regarded as a class of the Y-type and X-type with the corrugation angle of 45° and 60°, respectively. The PACK-1300XY constructed 12 sheets, six sheets with corrugation angles 60° and 45°. The completed details of PACK-1300XY are presented in Table 1.

**Table 1:** Key structural features of the PACK-1300XY during the simulation analyses.

Packing ID	a_p_ (1/m)	Void fraction (–)	Corrugation Specification	
			Angle	Height (mm)
PACK-1300XY	1300	0.91	45° and 60°	2

To elaborate on the accuracy of our results, we have employed the 3D tetrahedral cell. In addition, to verify the independence between the grid and HETP, several different mesh densities, including 1,520,000, 3,520,000, 5,230,000, and 13,680,000, have been used, see Table 2. This table confirms a slight improvement in the relative error (~ 1%) by reducing the element size from 0.2 to 0.1 mm. Consequently, 0.2 mm seems a proper element size for discretizing the model.

**Table 2:** Dependency of the HETP on the element size during the simulation analyses.

Element size (mm)	0.1	0.2	0.3	0.4
HETP (cm)	7.30	7.36	7.60	8.12

Two-phase flow mass transfer efficiency, dry/wet pressure drop, and isopropanol/methanol binary mixtures are investigated during the hydrodynamic inspection of the considered packing. The properties of this binary system have been presented in Table 3. By considering these parameters, at the top section of the column, we set the inlet velocity for the boundary condition and the pressure outlet in the outlet condition. It should be noted that careful considerations should be applied for the correct values for volume fraction, mass fraction of the methanol in each phase, turbulent quantities, and velocity component at the boundary conditions. Indeed, no-slip boundary conditions at the wall, $k - \omega$ turbulent model was used for both gas and liquid streams.

**Table 3:** The properties of the material (liquid density, liquid viscosity, liquid diffusion coefficient, gas density, gas viscosity, gas diffusion coefficient and surface tension) used for calculating the HETP in the binary system.

System	$\rho_{L}$ $\left( {{\text{kg/m}}^{3} } \right)$	$\mu_{L}$ $\left( {{\text{Ns/m}}^{2} } \right)$	$D_{L}$ $\left( {{\text{m}}^{2} /{\text{s}}} \right)$	$\rho_{G}$ $\left( {{\text{kg/m}}^{3} } \right)$	$\mu_{G}$ $\left( {{\text{Ns/m}}^{2} } \right)$	$D_{G}$ $\left( {{\text{m}}^{2} /{\text{s}}} \right)$	$\sigma$ $\left( {{\text{N}}/{\text{m}}} \right)$
Isopropanol/methanol	771	0.00033	$3.5 \times 10^{ - 9}$	1.33	0.0000106	$9.6 \times 10^{ - 6}$	0.018

The SIMPLE (semi-implicit method for pressure-linked equations) algorithm (the finite volume method) is used to compute the coupling between the pressure and velocity in the numerical stage. Also, the equations related to turbulent kinetic energy and momentum by the second-order upwind discretization. The commercial software package of CFX-18 has been employed for the computational simulations (the residual error of 0.0001 is considered for justifying the convergence).

## Results and discussion

### Inspection of the hydrodynamics behavior

#### Pressure drop, dry phase

The dry pressure drop alongside the column is a prominent parameter^73^, its effectiveness should also be considered another important characteristic to estimate the wet pressure drop. Moreover, the F-factor defined in Eq. (12) shows the loudness of gas in the packed bed.

12 $$F - factor = u_{g} = \sqrt {\rho_{g} }$$

**Figure 2:**
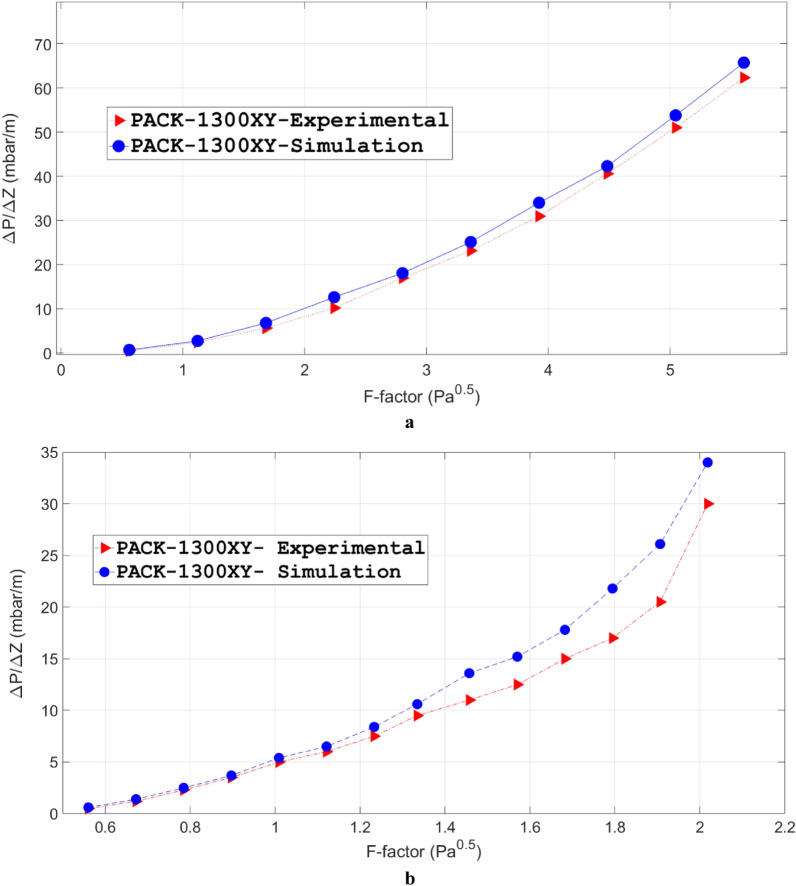
The experimental measurements (red triangle) and simulation results (blue circle) for the dry (**a**) and wet (**b**) pressure drop of the PACK-1300XY as a function of F-factor.

In this study, the dry pressure drop and flow field of the considered packing (i.e., PACK-1300XY) are calculated in the steady-state mode. The results are then validated by the experimental measurements in the literature^50^. Moreover, to confirm the trustworthiness and strength of the performed numerical calculations procedure, the pressure drop was applied between the entry and effluent gas streams. All these criteria led to a convergence precision of 1 × 10^–6^ for the investigation of the velocity effect on the pressure drop. Figure 2a indicates the comparison between the simulations results and experimental measurements versus F-factor for PACK-1300XY. In addition to the good consistency between simulations and experimental results, one can observe increasing velocity for the empirical correlation and computational results when the pressure drop was increased. Moreover, the results showed a mean relative deviation of ~ 14% for the numerical findings.

As shown in Fig. 2a, due to the gas phase distribution in the whole of packings, there are always lower amounts for simulations results than the empirical values^73^. Another reason for this behavior could be related to the fact that there is a uniform distribution for the gas phase in the packed bed, which would cause a reduction for pressure drop resulting from computational works in comparison to experimental measurements.

#### Pressure drop, wet phase

**Figure 3:**
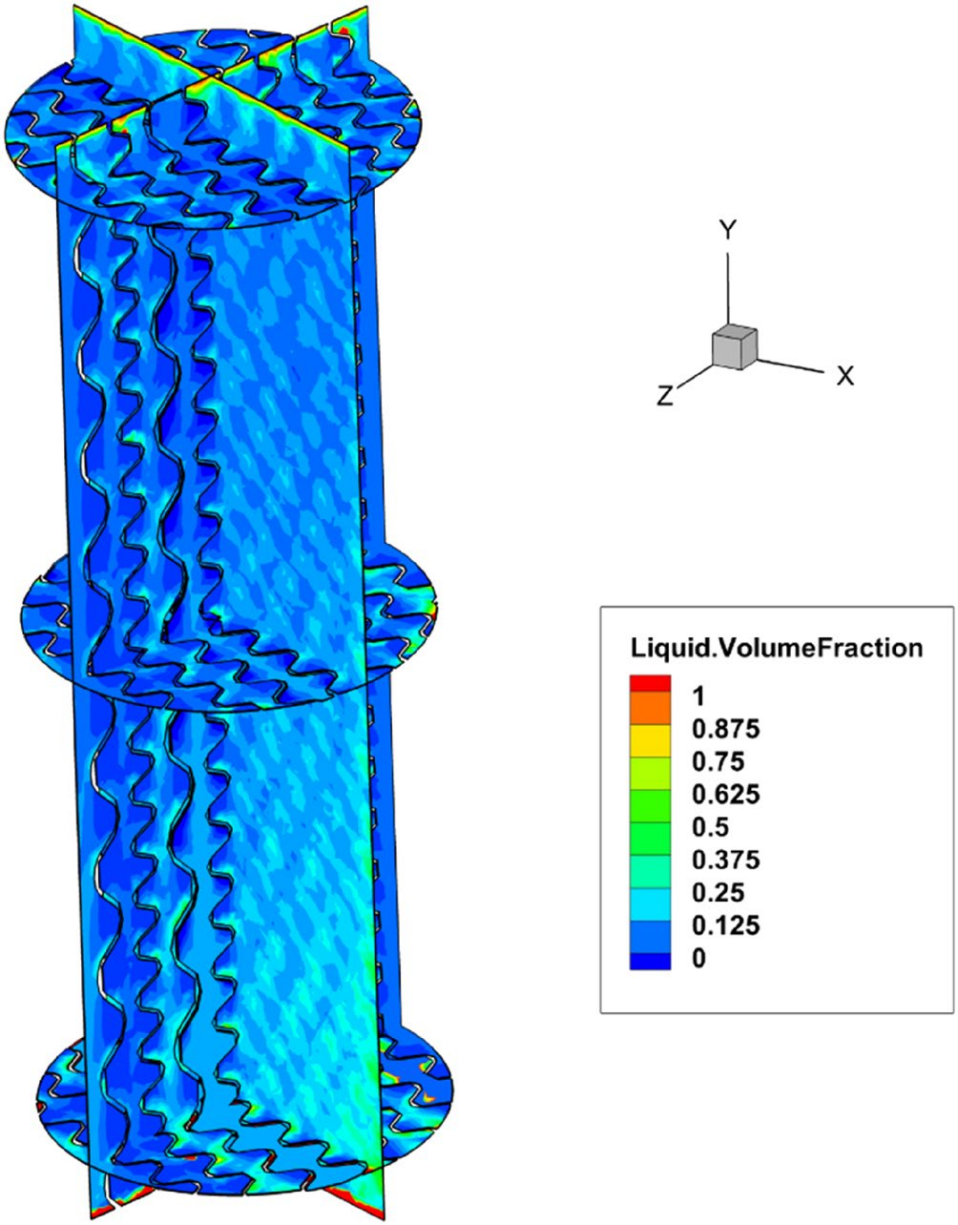
Liquid distribution around the PACK-1300XY obtained by the three-dimensional modeling (liquid flow rate = 7.5 m^3^/m^2^ h, F-factor = 1.2 Pa^0.5^).

The liquid volume fraction profile has been calculated for the binary air–water system along the packed bed and is shown in Fig. 3. This figure illustrates that the simulated structured packing has been covered with a thin layer of water. Furthermore, to better understand the relationship between the flow rate and pressure drop of gas, we compared the simulation findings and experimental measurements for the two-phase pressure drop of the binary system of air and water system, which has been provided in Fig. 2b. It could be concluded that when the rate of liquid flow increased, we observed an increasing trend in the gas pressure drop by fixing the gas velocity. This phenomenon would be explained because the decrease in the free cross-section can reduce the movement possibility of gas flow. Furthermore, the mean relative deviations between the computational results and experimental measurements were around 26% for liquid flow rate = 7.5 m^3^/m^2^ h; F-factor = 1.2 Pa^0.5^. It could also be concluded that at the higher gas velocities there is a tendency to underestimate the pressure drop for the model at the greater gas velocities. It could be clarified by a variety of chief phenomena which the key impact on the pressure drop, although these amounts have been ignored in the computational methods, including liquid back mixing, flow channeling, and rough gas/liquid-phase distribution. In conclusion, a rough flow distribution is necessary to achieve a more precise estimation for the two-phase pressure drop. The outputs of this study showed a mean relative deviation of ~ 16% for numerical data.

**Figure 4:**
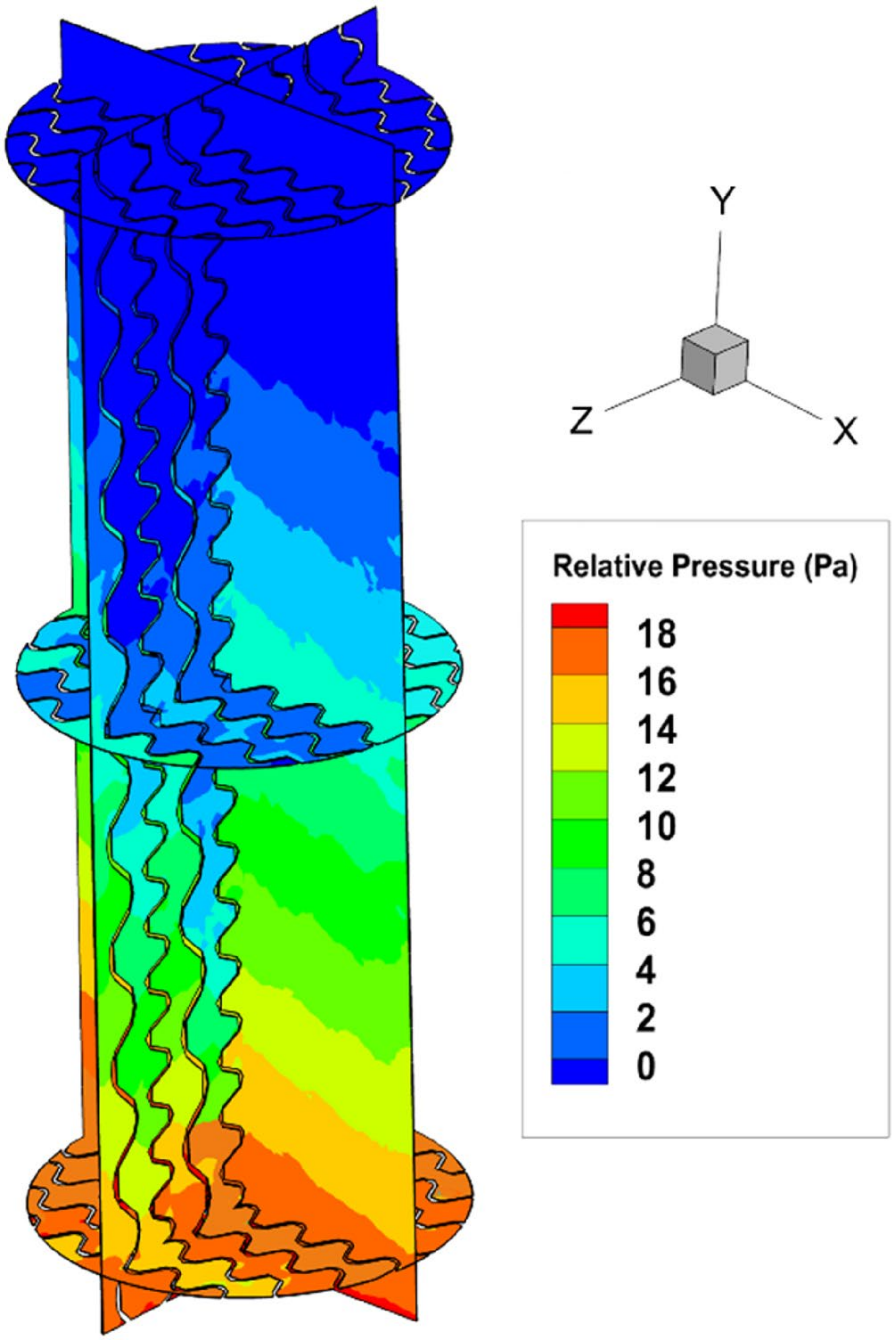
The pressure profile at the inside of the PACK-1300XY (F-factor = 1.2 Pa^0.5^, liquid flow rate = 7.5 m^3^/m^2^ h).

Based on Fig. 4 which is the distribution of the pressure around the packing, the wet pressure drop along the PACK-1300XY is ~ 190 Pa/m.

### Investigating the mass transfer efficiency

As mentioned previously, the HETP parameter is an important characteristic to explain the packed column’s separation efficiency^26^. Moreover, the concentration and velocity profiles along the packed bed height are needed to estimate the mass transfer efficiency of a distillation column equipped with structured packings. Therefore, it is vital to achieve the velocity distribution and the operational conditions for the calculation of the HETP^74^.

**Figure 5:**
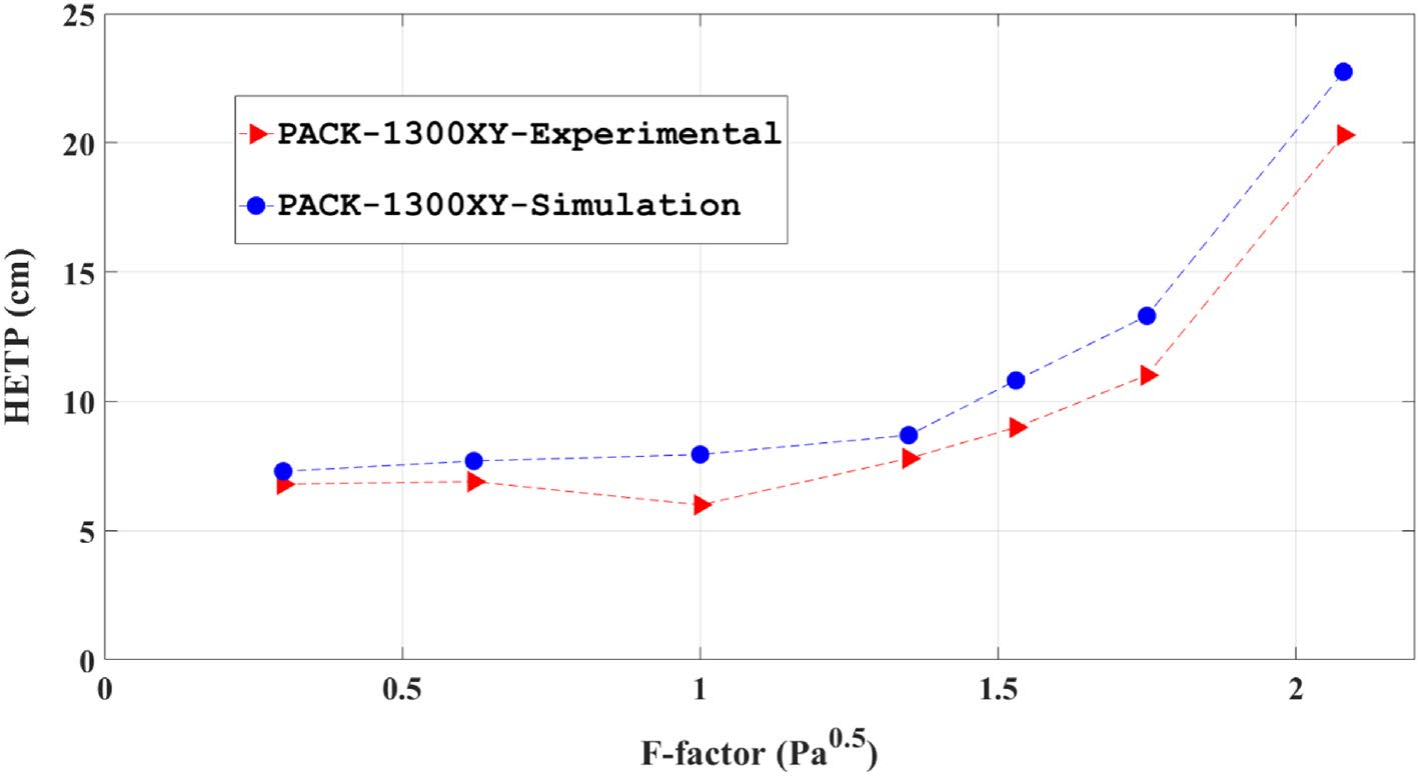
The experimental measurements (red triangle) and numerical predictions (blue circle) of the HETP of the PACK-1300XY.

Figure 5 compares the obtained mass transfer efficacy by the computational and experimental works for the PACK-1300XY. The comparison is made by monitoring the HETP of PACK-1300XY versus the gas F-factor. In addition to the expected trends of the HETP for the structured packings provided in the literature^75–77^, it could be observed in Fig. 5. Moreover, there is a gradual increase for HETP from 6 to 22 cm by increasing the amounts of 0.4 to 2.1 (kg/m^3^)^0.5^ m/s. Another finding from this figure is the difference between the computational and experimental results increased by increasing the F-factor, although there is a similar trend between them^49^. The computational results are verified by empirical findings^50^ and also the HETP parameter is the major result of these trials and estimates the column height. The possibility that the mass transfer across the interface might be influenced by interfacial effects. This phenomenon could be resistant to mass transfer and affected the concentration of outlet material^39–41^.

Similar to dry pressure drop behavior, there is a lower amount for computational results of HETP in various F-factor values in comparison to the empirical results^58^ as presented in Fig. 5. The reason for this behavior would be related to the assumption of uniform distribution for both the liquid and gas phases in computational technique which increases the mass transfer area and consequently reduction for the HETP values and increases mass transfer rates^48,58,78^. It should be noted that the average relative deficiency of around 17% was observed for numerical data in this study.

**Figure 6:**
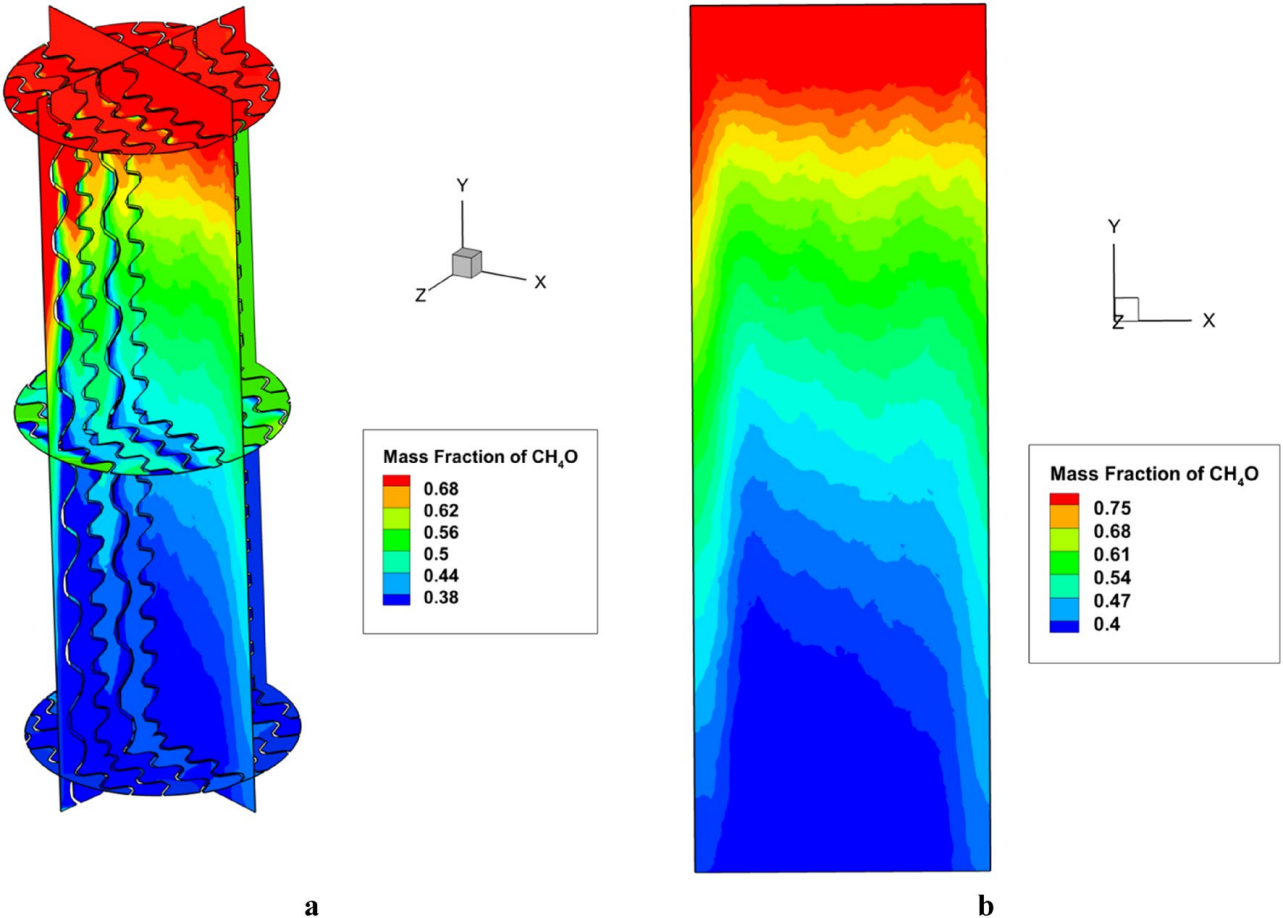
(**a**) The mass fraction contour of the methanol liquid phase and (**b**) two-dimensional mass fraction contour of methanol liquid phase.

Figure 6a presents the mass fraction contour for the liquid phase of CH_3_OH for the PACK-1300XY at F-factor = 1.6 m/s (kg/m^3^)^0.5^ for different horizontal and vertical segments. Moreover, the two-dimensional mass transfer contour according to the mass fraction of CH_3_OH has been depicted in Fig. 6b which there is an extreme alter for the concentration of CH_3_OH alongside both horizontal and vertical sections.

## Conclusion

This study successfully investigated several key hydrodynamic features of the innovative wire gauze structured packing, namely PACK-1300XY applying the CFD-based simulations. The focus is concentrated on the investigation of the pressure drop (dry and wet phases), mass transfer efficiency, and height equivalent to a theoretical plate. The HETP which is an important factor for mass transfer efficiency was studied for the isopropanol/methanol system. The three-dimensional CFD simulations technique with the Eulerian–Eulerian multiphase scheme has been applied in this regard. The results approved that the mean relative deviation between the computational predictions and experimental results are around 17% (for the mass transfer efficiency), and 14% and 16% for the dry and wet pressure drop, respectively. Therefore, the simulation method could be effectively used as a reliable tool for the simulation of these devices.
